# Immunogenicity and Vaccine Potential of InsB, an ESAT-6-Like Antigen Identified in the Highly Virulent *Mycobacterium tuberculosis* Beijing K Strain

**DOI:** 10.3389/fmicb.2019.00220

**Published:** 2019-02-12

**Authors:** Woo Sik Kim, Hongmin Kim, Kee Woong Kwon, Sang-Nae Cho, Sung Jae Shin

**Affiliations:** ^1^Department of Microbiology, Institute for Immunology and Immunological Disease, Brain Korea 21 PLUS Project for Medical Science, Yonsei University College of Medicine, Seoul, South Korea; ^2^Advanced Radiation Technology Institute, Korea Atomic Energy Research Institute, Jeongeup, South Korea

**Keywords:** *Mycobacterium tuberculosis*, Beijing genotype, ESAT-6, InsB, subunit vaccine

## Abstract

Our group recently identified InsB, an ESAT-6-like antigen belonging to the Mtb9.9 subfamily within the Esx family, in the *Mycobacterium tuberculosis* Korean Beijing strain (Mtb K) via a comparative genomic analysis with that of the reference Mtb H37Rv and characterized its immunogenicity and its induced immune response in patients with tuberculosis (TB). However, the vaccine potential of InsB has not been fully elucidated. In the present study, InsB was evaluated as a subunit vaccine in comparison with the most well-known ESAT-6 against the hypervirulent Mtb K. Mice immunized with InsB/MPL-DDA exhibited an antigen-specific IFN-γ response along with antigen-specific effector/memory T cell expansion in the lungs and spleen upon antigen restimulation. In addition, InsB immunization markedly induced multifunctional Th1-type CD4^+^ T cells coexpressing TNF-α, IL-2, and IFN-γ in the lungs following Mtb K challenge. Finally, we found that InsB immunization conferred long-term protection against Mtb K comparable to that conferred by ESAT-6 immunization, as evidenced by a similar level of CFU reduction in the lung and spleen and reduced lung inflammation. These results suggest that InsB may be an excellent vaccine antigen component for developing a multiantigenic Mtb subunit vaccine by generating Th1-biased memory T cells with a multifunctional capacity and may confer durable protection against the highly virulent Mtb K.

## Introduction

Tuberculosis (TB), caused by *Mycobacterium tuberculosis* (Mtb), remains a major public health threat worldwide as the top infectious disease in terms of morbidity and mortality (WHO, 2017). Despite the global use of *Bacillus* Calmette-Guerin (BCG) vaccination and available TB treatments, TB reportedly showed an incidence of 10.4 million cases and caused 1.7 million deaths in 2016 (WHO, 2017). Although the prevention of TB is the most effective control measure for reducing the incidence of TB, the protective efficacy of BCG, which is the only approved vaccine for TB (Nemes et al., 2018), is thought to be insufficient to protect against pulmonary TB and latent infection, and its highly variable results among different geographical locations indicate that Mtb genotypes with different virulence levels might be dominant in different regions (Pitt et al., 2013).

To develop new prophylactic vaccines capable of replacing or improving the BCG vaccine, researchers have moved many vaccine candidates into the clinical phase (Kaufmann et al., 2017). The identification and discovery of novel antigens is the initial and important step in new vaccine development (Singh et al., 2014). Importantly, an understanding of antigenic variation and the differential virulence levels of clinically prevalent Mtb strains is one of the factors considered in TB vaccine development (Ernst, 2017; Chae and Shin, 2018; Chiner-Oms et al., 2018). In addition, studies of newly emerging strains displaying a wide spectrum of virulence and fitness have been considered as valuable for developing new vaccines, as screening vaccines with laboratory-adapted strains have been regarded as one possible limitation in the current field (Henao-Tamayo et al., 2015). In particular, the Mtb Beijing genotype is highly dominant in East Asian countries, and the rate of isolation of strains belonging to the Mtb Beijing family has increased worldwide, which indicates that the BCG vaccine might provide a relatively low level of protection against these strains (Abebe and Bjune, 2006; Kremer et al., 2009). Furthermore, epidemiological studies have suggested that extensive and continuous BCG vaccination may be one of the forces causing the emergence of the Beijing genotype (Abebe and Bjune, 2006), indicating that the global control of Mtb Beijing strains is important due to their association with drug resistance and their ability to evade BCG-conferred vaccine efficacy (Kremer et al., 2009). Furthermore, the failure of the MVA85A vaccine trial may have occurred because it was tested not against any clinical strains but only against laboratory-adapted strains without considering the prevalent local strains in the region of clinical trials even though MVA85A was extensively tested in animal settings (Groschel et al., 2017). In this context, greater attention to the varying fitness of Mtb strains throughout the regions should be preferentially required for the development of a vaccine and testing of its efficacy. Thus, the protective efficacy of new TB vaccine candidates should be tested against the prevailing local strains, such as Mtb Beijing strains, in addition to the laboratory-adapted strains (van Soolingen et al., 1995).

In this regard, we previously characterized the genetic features of the Mtb Korean Beijing strain (Mtb K) causing a high school TB outbreak in South Korea via whole-genome sequencing (Han et al., 2015) and a comparative genomics approach to analyze the reference Mtb H37Rv strain (GenBank accession no. NC_000962) and Mtb K (GenBank accession no. CP007803.1). Interestingly, we identified MTBK_24790 (GenBank: AIB49023.1, hereafter referred to as InsB according to our previous study) (Park et al., 2014), an ESAT-6-like family protein encoded within the 5.7 kb gene cluster specifically inserted into the genome of Mtb strain K. Further analysis of sequences homologous to InsB by protein BLAST analysis^1^ revealed that an identical protein in the reported Mtb genome was found only in CDC1551, which was well characterized as a highly transmissible and virulent strain during TB outbreaks in the United States (Valway et al., 1998). In addition, our previous studies demonstrated that the recombinant InsB of Mtb K demonstrated a potential to improve the sensitivity of immunodiagnosis in both humans and mice (Park et al., 2014; Hur et al., 2016) by inducing an immune response differing from that induced by ESAT-6.

The *esx* family consists of 23 members, and the corresponding Esx proteins are involved in host-pathogen interactions during Mtb infection (Skjot et al., 2002). In addition, these secreted Esx family proteins are considered to be the most immunodominant antigens recognized by the host immune system and have therefore been exploited to develop vaccines and immunodiagnostic tools for TB control (Groschel et al., 2016). Among the Esx family proteins, the Mtb9.9 subfamily proteins, with 94 amino acids and a molecular size of approximately 9.9 kDa, comprises five members: EsxN, EsxI, EsxL, EsxO, and EsxV (Knudsen et al., 2014; Villarreal et al., 2014). Although the function of Mtb9.9 subfamily antigens remains unknown, Peng et al. (2012) reported that the Mtb9.9 subfamily proteins are immunogenic in C57BL/6 mice by inducing humoral and Th1-type cellular memory responses, suggesting that they may serve as potent subunit vaccine candidates. Moreover, Villarreal et al. (2014) demonstrated that the strongest CD4^+^ T-cell immune responses were observed for EsxO antigen and EsxV, which are located in region of difference (RD) 7 and RD9 and are absent in BCG; EsxV was the most prominent antigen to induce CD8^+^ T cell immunity among the Mtb9.9 subfamily antigens. Furthermore, Mahmood et al. (2011) reported that EsxV antigen induced a strong Th1 immune response; reduced the bacterial burden in the lungs of Mtb-infected mice; caused increased IFN-γ, IL-12 and IgG2a levels; and can thus serve as a subunit vaccine candidate. Therefore, these immunogenic antigens may be useful for designing and developing more effective TB vaccines.

Interestingly, a homologous protein sequence analysis of InsB revealed that InsB belongs to the Mtb9.9 protein subfamily by showing >95% protein identity of InsB to all members of the Mtb9.9 protein subfamily (EsxI, EsxL, EsxN, EsxO, and EsxV) (Park et al., 2014), and Mtb K has one more copy of the region of the InsB cluster with an unknown function. In addition, despite their low mass, the Mtb9.9 protein antigens contain at least five distinct T cell epitopes. Moreover, they are the specific proteins of Mtb that are not detected in BCG (Peng et al., 2012). In addition, biased Th1-type cellular and humoral memory responses to the Mtb9.9 protein family have been shown in animal studies, and multiple cytokines and multicytokine-producing T cells are induced by these proteins. Thus, we should be able to evaluate their contributions to the development of diagnostic tools and vaccines for TB control if a new Esx antigen or its epitopes are identified (Peng et al., 2012).

Our group has searched for optimal vaccine antigen combinations aimed at developing a multiantigenic vaccine and has attempted to characterize well-known or less well-known antigens *in vitro* and *in vivo* (Kim et al., 2013; Choi et al., 2015, 2017; Kwon et al., 2017). Thus, the primary goal of our current study was to investigate the vaccine potential of InsB based on comparative genomic analysis selection as a single antigen against Mtb K, which remains predominantly isolated in South Korea (Kang et al., 2010). In the present study, the immunogenicity and the potential protective efficacy of InsB and ESAT-6 as subunit vaccines were compared in a head-to-head manner using a Mtb Beijing strain challenge model.

## Materials and Methods

### Animals and Ethics Statement

Specific pathogen-free female C57BL/6 mice (6–7 weeks of age) were purchased from Jackson Laboratory (Bar Harbor, ME, United States) and maintained under barrier conditions in a BL-3 biohazard animal facility of the Laboratory Animal Research Center at Yonsei University College of Medicine (Seoul, South Korea). This study was carried out in accordance with the recommendations of the Korean Food and Drug Administration (KFDA), the Korean Institutional Animal Ethical Committee. The animal experimental protocols used in this vaccine study were approved by the Korean Institutional Animal Ethical Committee (Permit Number: 2014-0197-3).

### Cloning and Purification of Recombinant InsB Protein

Recombinant ESAT-6 and InsB proteins were purified as previously described (Park et al., 2014; Hur et al., 2016). Briefly, Mtb K was grown in Sauton’s media at 37°C, and its genomic DNA was prepared by using N-acetyl-N,N,N-trimethyl ammonium bromide (CTAB) buffer. PCR was performed to amplify the InsB sequence with a primer set (F: 5′-TTGCATATGACGATCAATT-ATCAGTTCGG-3′; R: 5′- GCGGATCCAGCCCAGCT-GGAACCCACT-3′). The PCR product was confirmed and inserted into pET11a_KB with the NdeI and BamH1 restriction enzymes. The vector DNA was transferred to *Escherichia coli* DH5α, and plasmids were then extracted using an Expin Plasmid Purification Kit (GeneAll, Seoul, South Korea). This recombinant protein was transferred again to *E. coli* BL21 (DE3) and purified with Ni-NTA resin for histidine affinity chromatography using AKTA fast protein liquid chromatography (FPLC) with a Mono Q anion exchange column. Following separation by FPLC, proteins were confirmed by SDS-PAGE.

### Antigen Immunization and Mtb K Challenge in Mice

For the adjuvant control, mice were immunized subcutaneously 3 times at 3-week intervals with dimethyldioctadecylammonium (DDA) liposomes (50 μg) containing monophosphoryl lipid-A (MPL, 5 μg). MPL and DDA were purchased from Sigma-Aldrich (St. Louis, MO, United States). MPL was mixed in distilled water, and this mixture was heated in a water bath at 65°C for 30 s and then sonicated for 30 s, and this heating and sonication steps were repeated three times. MPL was subsequently mixed with DDA, which was dissolved in distilled water, at a 2:1 (MPL:DDA) ratio immediately before use. For ESAT-6 and InsB immunization, mice were immunized subcutaneously 3 times at 3-week intervals with a formulation containing each antigen (InsB, 5 μg; ESAT-6, 5 μg) and MPL-DDA (this adjuvant concentration was the same as that used for the adjuvant control groups). The BCG-vaccinated groups (as a positive control for the efficacy testing experiment for the TB vaccine) were subcutaneously vaccinated with 2.0 × 10^5^ CFU of BCG Pasteur 1173 P2 at the time of the 2nd immunization using the antigens. Three weeks after the last immunization, adjuvant controls, antigen-immunized mice and BCG-vaccinated mice were aerogenically infected with Mtb K as previously described (Kim et al., 2013). Briefly, the mice were exposed to Mtb K for 60 min in the inhalation chamber of an airborne infection apparatus calibrated to deliver a predetermined dose (Glas-Col, Terre Haute, IN, United States). To confirm the initial bacterial burden, four mice were euthanized one day later, and an analysis of these mice revealed that approximately 200 CFUs were delivered to the lungs of each mouse.

### Splenocyte and Lung Cell Preparation

Single-cell suspensions from lung and spleen were prepared as follows. The spleen and lung from mice of each group were homogenized. Spleen homogenates were filtered through a 40 μm cell strainer (BD Bioscience, San Diego, CA, United States) in RPMI medium supplemented with 2% fetal bovine serum (FBS, Biowest, Nuaillé, France) using a sterile 10 mL syringe. The lung cells were prepared as previously described (Kim et al., 2015). Briefly, the lung homogenates were incubated in 3 mL of cellular dissociation buffer RPMI medium (Biowest) containing 0.1% collagenase type IV (Worthington Biochemical Corporation, NJ, United States) and 1 mM CaCl_2_ and 1 mM MgCl_2_) for 15 min at 37°C, and then lung cells were filtered through a 40 μm cell strainer in RPMI medium supplemented with 2% fetal bovine serum using a sterile 10 mL syringe. The erythrocytes were lysed using red blood cell lysis buffer (Sigma-Aldrich) for 5 min at room temperature, and then single-cells were washed twice with RPMI medium supplemented with 2% FBS.

### Measurement of IFN-γ Production

Three weeks after the final immunization of antigens, single-cell suspensions (1 × 10^6^/mL) from the lung and spleen of adjuvant-, BCG-, and antigen-immunized mice were stimulated with purified proteins (InsB, 2 μg/mL; ESAT-6, 2 μg/mL) for 24 h at 37°C. IFN-γ cytokine levels were analyzed in the culture supernatant via sandwich enzyme-linked immunosorbent assay (ELISA) according to the manufacturer’s protocol. Additionally, single-cell suspensions were stimulated with purified proteins (InsB; 2 μg/mL, ESAT-6; 2 μg/mL) for 12 h at 37°C in the presence of GolgiStop (eBioscience, San Diego, CA, United States), and then the cells were stained with Live/Dead Stain (InvivoGen, San Diego, CA, United States) and with anti-CD4 (PerCp-Cy5.5, eBioscience), anti-CD8 (APC-Cy7, eBioscience), and anti-CD3 (BV421, eBioscience) antibodies for 30 min at 4°C. Next, the stained cells were fixed and permeabilized using a Cytofix/Cytoperm kit (BD Biosciences, San Jose, CA, United States) according to the manufacturer’s protocol and then stained with anti-IFN-γ (PE, eBioscience). The cells were analyzed with a FACSVerse flow cytometer using FlowJo software.

### Analysis of Antigen-Specific Multifunctional T Cells

Single-cell suspensions from the lung and spleen of Mtb K-infected mice at 3 weeks after the final immunization and at 4 and 9 weeks postchallenge were stimulated with purified proteins (InsB; 2 μg/mL, ESAT-6; 2 μg/mL) for 12 h at 37°C in the presence of GolgiStop. The cells were stained using a Live/Dead Fixable Aqua Dead Cell stain kit (BV510, Invitrogen) and with anti-CD3 (FITC, eBioscience), anti-CD4 (PerCp-Cy5.5), anti-CD8 (APC-Cy7), anti-CD44 antibodies (eFluor 450, eBioscience) for 30 min at 4°C and then fixed and permeabilized using a Cytofix/Cytoperm kit according to the manufacturer’s protocol. Next, the cells were stained with anti-IFN-γ (PE, eBioscience), anti-TNF-α (APC, eBioscience) and anti-IL-2 (PE-Cy7, eBioscience) for 30 min at 4°C. Cells stained with the appropriate isotype-matched immunoglobulins, which included rat IgG2b kappa (eFluor 450, eBioscience), mouse IgG1 kappa (PE, eBioscience), rat IgG1 kappa (APC, eBioscience), and rat IgG2b kappa (PE-Cy7, eBioscience) as the isotype controls for anti-CD44, IFN-γ, TNF-α, and IL-2 Abs, respectively, were used for discriminating cytokine-producing cells. The cells were analyzed with a FACSVerse flow cytometer using FlowJo software. To reduce false positives of cytokine-producing populations caused by non-specific staining, fewer than 10 cells in lung and 15 cells in spleen were considered as zero.

### Evaluation of Bacterial Burden and Lung Inflammation

The numbers of viable bacteria in the lung and spleen of Mtb K-infected mice were obtained for CFU counts. Briefly, each bacterial count was determined by plating serial dilutions of the organ homogenates onto Middlebrook 7H10 agar (Becton Dickinson, Franklin Lakes, NJ, United States) supplemented with 10% OADC enrichment medium until the late-exponential phase. After 3 weeks of incubation at 37°C, the numbers of colonies were then counted, and the values are reported as the mean log_10_ CFUs ± SDs per gram of lung and spleen tissues. The lung samples collected for histopathology were preserved overnight in 10% normal buffered formalin, embedded with paraffin, cut into 4–5-mm sections, and stained with hematoxylin-eosin (H&E). Then, the severity of lung inflammation was examined using the ImageJ program (National Institutes of Health, United States) as previously described (Jeon et al., 2011; Kwon et al., 2017). In brief, three images of each lung section (six mice/group) were obtained at 20× magnification using a Nikon ECLIPSE Ci (Nikon Corporation, Japan). After color deconvolution of each image into the red channel using the ImageJ program, the inflamed and non-inflamed areas could be observed as dark and light areas, respectively. Image analysis was independently conducted at each indicated time point. By discriminating inflamed areas from non-inflamed areas, the relative mean percentages of inflammation in the lung images from each group were obtained. The data are displayed using box-and-whisker plots.

### Statistical Analyses

Statistical analyses were conducted using GraphPad Prism V5.0 (GraphPad Software, San Diego, CA, United States). The differences between two groups were analyzed using unpaired Student’s *t*-test. One-way ANOVA followed by Tukey’s multiple comparison test was used to analyze differences between more than two groups. All the values are expressed as the means (±SDs). Differences with ^∗^*p* < 0.05, ^∗∗^*p* < 0.01 or ^∗∗∗^*p* < 0.001 were considered to be statistically significant.

## Results

### Expression of Recombinant InsB Protein and the Antigen-Specific IFN-γ Response

**FIGURE 1:**
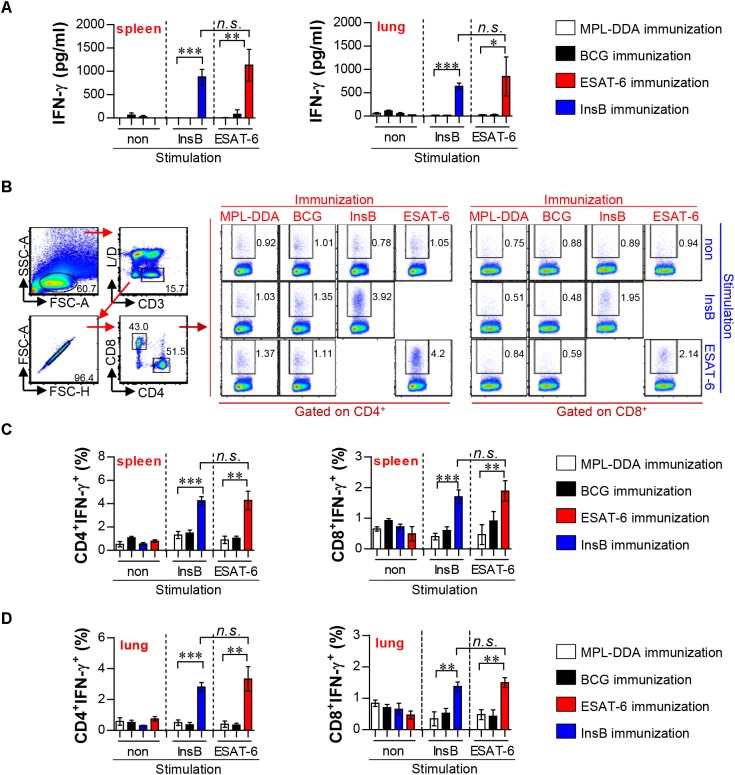
Immunogenicity in the lung and spleen of ESAT-6- and InsB-immunized mice. **(A)** At 3 weeks after the last immunization, mice (*n* = 6 mice/group) were immunized with ESAT-6, InsB, adjuvant alone (MPL-DDA), or BCG, and their splenocytes and lung cells were stimulated *in vitro* with no antigen, with ESAT-6, or with InsB for 24 h at 37°C. IFN-γ secretion by spleen and lung cells in response to ESAT-6 and InsB stimulation was analyzed by ELISA. **(B–D)** Spleen and lung cells were stimulated *in vitro* with no antigen, with ESAT-6, or with InsB in the presence of GolgiStop for 12 h at 37°C. The representative plot for IFN-g-producing CD4^+^ and CD8^+^ T cells in every vaccinated group of spleen is presented in **(B)**. The percentages of IFN-γ-producing CD4^+^ and CD8^+^ T cells of spleen **(C)** and lung **(D)** were analyzed by flow cytometry. The mean ± SDs (*n* = 6 mice/group) shown are representative of two independent experiments. Data from one of two independent experiments are shown. ^∗^*p* < 0.05, ^∗∗^*p* < 0.01, ^∗∗∗^*p* < 0.001. *n.s.,* no significant difference.

The *InsB* gene was cloned and expressed as described previously (Park et al., 2014). CD4^+^ Th1 cells and type-1 cytokines are essential for resistance to Mtb infection; IFN-γ is a key CD4^+^ Th1 cell-derived cytokine and could serve as a marker of recognition by the host immune system (Coler et al., 2018). Therefore, we first investigated the production of antigen-specific IFN-γ prior to challenge with an Mtb strain through ELISA (Figure 1A) and flow cytometry (Figure 1B–D). For this experiment, mice were immunized with InsB, and the control groups were immunized with BCG, ESAT-6, or MPL-DDA, which was used as an adjuvant. After final immunization, lung cells and splenocytes from all groups were stimulated with InsB and ESAT-6, respectively. When stimulated with antigens, lung and spleen cells of the InsB-immunized groups showed significantly increased IFN-γ production compared with those of the adjuvant control group (MPL-DDA); interestingly, this production showed a similar pattern (increased IFN-γ production) to those of the ESAT-6-immunized groups (Figure 1A). In addition, populations of IFN-γ-producing T cells (IFN-γ-producing CD4^+^ and CD8^+^) were analyzed in the spleen and lung cells of the immunized groups. As a consequence, increased frequencies of IFN-γ-producing CD4^+^ and CD8^+^ T cells were observed in the spleen (Figure 1B,C) and the lung (Figure 1D) of InsB- and ESAT-6-immunized groups. However, InsB- or ESAT-6-specific IFN-γ production was not observed in the BCG vaccine groups, which was expected because neither protein is included in the BCG vaccine (Figure 1A–D). These results indicate that InsB has immunogenic potential and that immunization with InsB induces a comparable Th1 immune response to that induced by ESAT-6 immunization.

### Multifunctional T Cells Induced in Mice Immunized With InsB Prior to Mtb Strain Challenge

**FIGURE 2:**
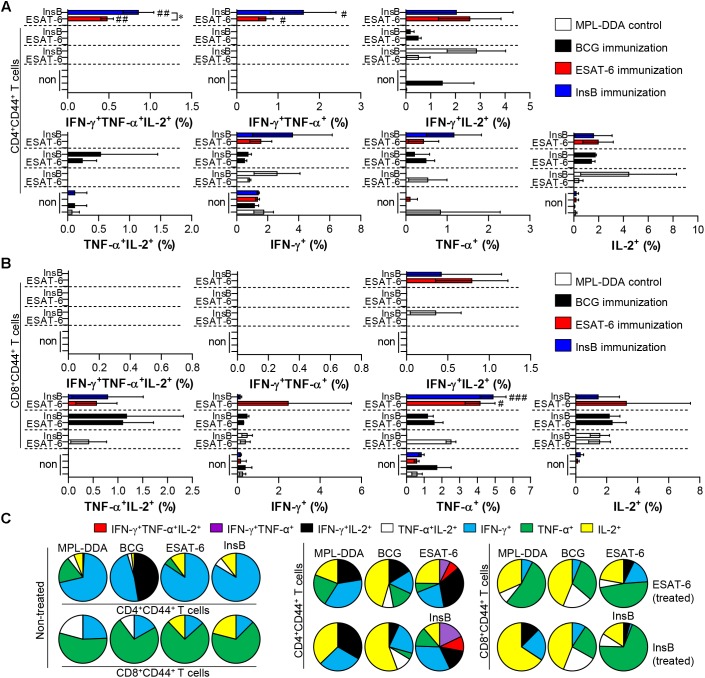
Induction of antigen-specific multifunctional T cells in the lung of ESAT-6- and InsB-immunized mice. At 3 weeks after the last immunization, lung cells from adjuvant (MPL-DDA)-, BCG-, ESAT-6-, or InsB-immunized mice (*n* = 6 mice/group) were stimulated *in vitro* with no antigen, with ESAT-6, or with InsB in the presence of GolgiStop for 12 h at 37°C. The percentage of antigen-specific CD3^+^CD4^+^CD44^+^ **(A)** and CD3^+^CD8^+^CD44^+^ **(B)** T cells producing IFN-γ, TNF-α, or IL-2 was measured according to the gating strategy shown in Supplementary Figure 1. The frequency of T cells producing each combination of cytokines is shown as the percentage of the specific cell type in the CD3^+^CD4^+^CD44^+^ or CD3^+^CD8^+^CD44^+^ T cell population. The mean ± SDs (*n* = 6 mice/group) shown are representative of two independent experiments. ^∗^*p* < 0.05 (InsB vs. ESAT-6-immunized group) and ^#^*p* < 0.05, ^##^*p* < 0.01, ^###^*p* < 0.001 (antigen-immunized groups vs. each antigen-treated MPL-DDA group). To reduce false positives of cytokine-producing populations caused by non-specific staining, fewer than 10 cells in lung and 15 cells in spleen were considered as zero. **(C)** Pie charts represent the mean frequencies of cells coexpressing IFN-γ, TNF-α, or IL-2.

Although immune correlates of protection against TB in humans have not yet been determined, multifunctional Th1-type T cells (that produce IFN-γ, TNF-α, and IL-2) are considered a major component contributing to protection in animal models (Flynn and Bloom, 1996; Flynn, 2004; Nandakumar et al., 2014; Karp et al., 2015; Coler et al., 2018). Therefore, we next investigated the frequency and proportion of antigen-specific single- (IFN-γ^+^-, TNF-α^+^-, IL-2^+^-producing T cells), double- (IFN-γ^+^TNF-α^+^-, IFN-γ^+^IL-2^+^-, TNF-α^+^IL-2^+^-producing T cells) or triple-positive multifunctional T cells (IFN-γ^+^TNF-α^+^IL-2^+^-producing T cells) after gating CD3^+^CD4^+^CD44^+^ and CD3^+^CD8^+^CD44^+^ cells by multicolor flow cytometric analysis (Supplementary Figure S1). At 3 weeks after the final immunization, lung cells and splenocytes of InsB-, ESAT-6- and BCG-immunized mice were restimulated with InsB and ESAT-6, respectively, and the MPL-DDA-injected group was used as the control. InsB- and ESAT-6-immunized groups showed higher levels of triple-positive and double-positive (IFN-γ^+^TNF-α^+^) CD3^+^CD4^+^CD44^+^ T cells in lung cells (Figure 2A,C) and splenocytes (Supplementary Figures S2A,C) than did the control group injected with MPL-DDA; interestingly, these cell types induced by InsB immunization were significantly increased to a similar extent to that induced by ESAT-6 immunization. For CD8^+^ T cells, triple-positive and double-positive (IFN-γ^+^TNF-α^+^ and IFN-γ^+^TNF-α^+^) CD3^+^CD8^+^CD44^+^ T cells were observed only in the spleens of InsB- and ESAT-6-immunized mice (Supplementary Figures S2B,C), but significant differences between InsB and ESAT-6 immunization were not reported. These results indicated that InsB is a potential candidate antigen for inducing an effective Th1 immune response.

### Protective Efficacy of Antigen Immunization Against Mtb K Infection With Respect to Bacterial Growth and Histopathology

**FIGURE 3:**
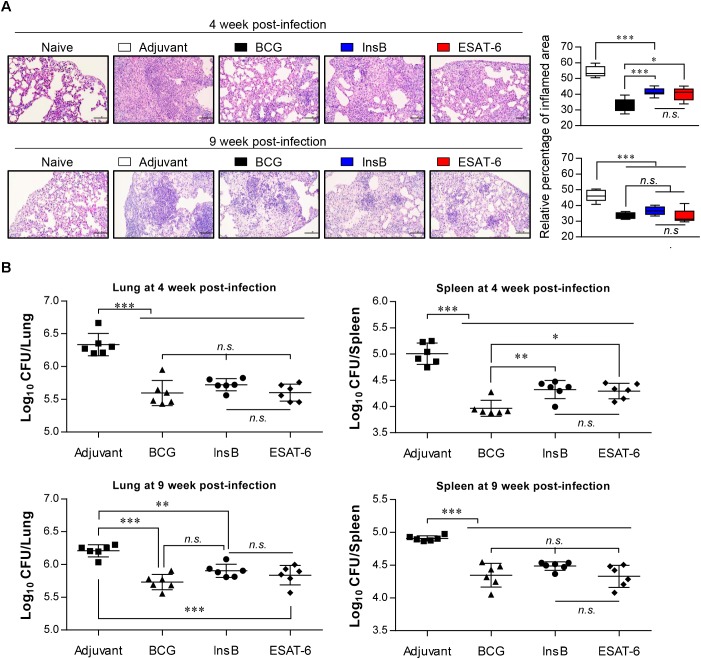
Protective efficacy of antigen immunization against Mtb K infection. (**A**) H&E-stained sections (*n* = 6 mice/group) of the lung from naive, adjuvant alone (MPL-DDA)-, BCG-, ESAT-6-, or InsB-immunized mice at 4 and 9 weeks after challenge with the aerosolized Mtb K strain (20X: Scale bar = 100 μm). The experimental results represent the relative percentages of inflamed area to unaffected area of lungs from uninfected mice as box-and-whisker plots. **(B)** Bacterial numbers in the lung and spleen of adjuvant-, BCG-, InsB-, or ESAT-6-immunized mice at 4 and 9 weeks after Mtb K challenge are shown. The mean estimated log_10_ CFUs ± SDs (*n* = 6 mice/group) shown are representative of two independent experiments. One-way ANOVA followed by Tukey’s multiple comparison test was used to evaluate the significance. ^∗^*p* < 0.05, ^∗∗^*p* < 0.01, ^∗∗∗^*p* < 0.001. *n.s.,* no significant difference.

Based on the immunological results of InsB immunization, we next measured the protective efficacy of InsB immunization against Mtb K challenge. Four weeks after the final immunization, mice were infected with Mtb K via the aerosol route. Four and nine weeks after Mtb K challenge, bacterial load and histopathological analysis were examined in the naive, MPL-DDA-injected control and in the BCG-, ESAT-6- and InsB-immunized groups. Hematoxylin and eosin (H&E) staining of lung tissues indicated that InsB immunization induced a reduction in granulomatous inflammation to a similar extent to that conferred by ESAT-6 immunization at 4 and 9 weeks postchallenge of Mtb K-infected mice. Moreover, this reduced lung inflammation was comparable to that of the BCG-immunized group at 9 weeks postchallenge (Figure 3A). In addition, InsB immunization resulted in a decreased bacterial burden in the lung and spleen, and this decrease was as great as that in the ESAT-6 or BCG-immunized groups; furthermore, these protective efficacies were sustained until 9 weeks postinfection (Figure 3B). These results suggest that InsB subunit vaccination may offer a protective efficacy similar to that of BCG or ESAT-6.

### Induced InsB-Specific Effective Multifunctional T Cell Immunity After Challenge With Mtb in the Spleen and Lung

**FIGURE 4:**
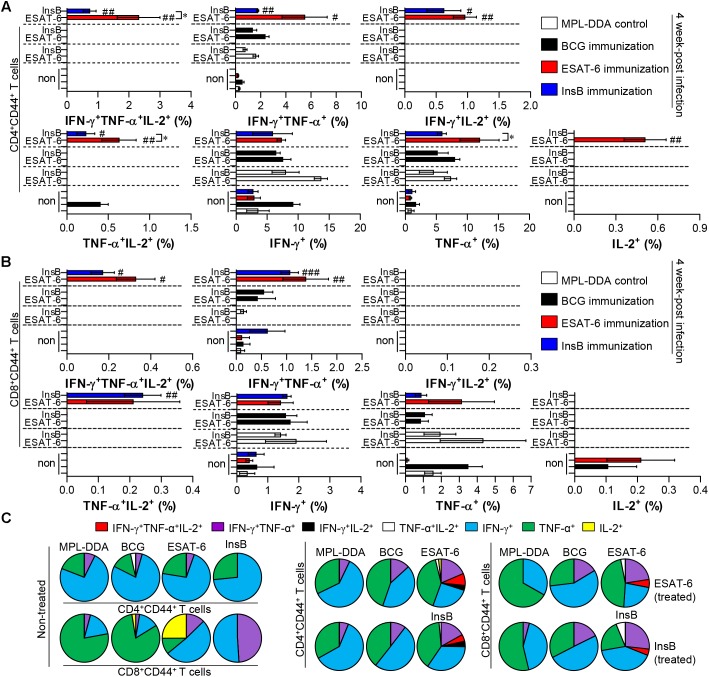
Induction of antigen-specific multifunctional T cells in the lung of ESAT-6- and InsB-immunized mice at 4 weeks postinfection. Four weeks postinfection, lung cells from adjuvant (MPL-DDA)-, BCG-, ESAT-6-, or InsB-immunized mice (*n* = 6 mice/group) were stimulated *in vitro* with no antigen, with ESAT-6, or with InsB in the presence of GolgiStop for 12 h at 37°C. The cell staining method for the multifunctional T cells is presented in the Materials and Methods section. The percentage of antigen-specific CD3^+^CD4^+^CD44^+^ **(A)** and CD3^+^CD8^+^CD44^+^ **(B)** T cells producing IFN-γ, TNF-α, or IL-2 was analyzed using flow cytometry. The frequency of T cells producing each combination of cytokines is presented as the percentage of the specific cell type in the CD3^+^CD4^+^CD44^+^ or CD3^+^CD8^+^CD44^+^ T cell population. The mean ± SDs (*n* = 6 mice/group) shown are representative of two independent experiments. ^∗^*p* < 0.05 (InsB vs. ESAT-6-immunized group) and ^#^*p* < 0.05, ^##^*p* < 0.01, ^###^*p* < 0.001 (antigen-immunized groups vs. each antigen-treated MPL-DDA group). To reduce false positives of cytokine-producing populations caused by non-specific staining, fewer than 10 cells in lung and 15 cells in spleen were considered as zero. **(C)** Pie charts represent the mean frequencies of cells coexpressing IFN-γ, TNF-α, or IL-2.

**FIGURE 5:**
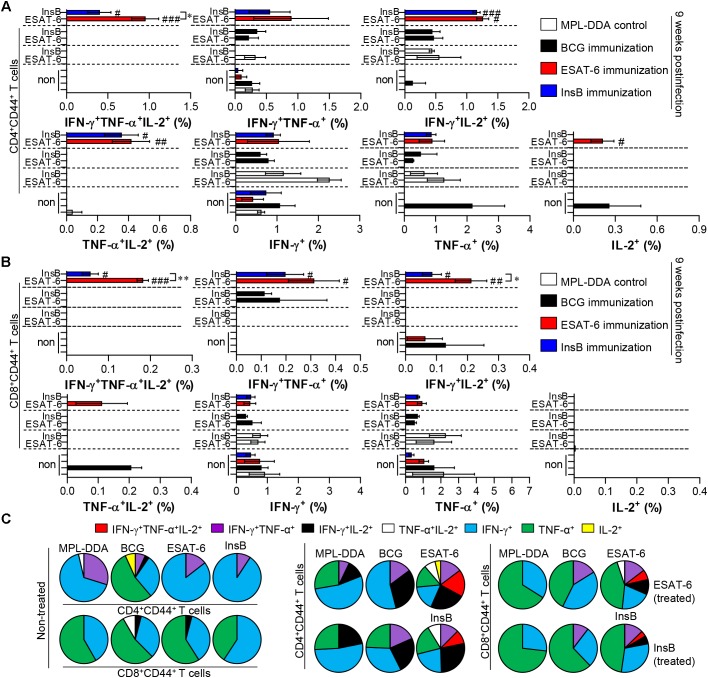
Induction of antigen-specific multifunctional T cells in the lung of ESAT-6- and InsB-immunized mice at 9 weeks postinfection. Nine weeks postinfection, lung cells from adjuvant (MPL-DDA)-, BCG-, ESAT-6-, or InsB-immunized mice (*n* = 6 mice/group) were stimulated *in vitro* with no antigen, with ESAT-6, or with InsB in the presence of GolgiStop for 12 h at 37°C. The percentage of antigen-specific CD3^+^CD4^+^CD44^+^ **(A)** and CD3^+^CD8^+^CD44^+^ **(B)** T cells producing IFN-γ, TNF-α, or IL-2 was analyzed using flow cytometry. The frequency of T cells producing each combination of cytokines is presented as the percentage of the specific cell type in the CD3^+^CD4^+^CD44^+^ or CD3^+^CD8^+^CD44^+^ T cell population. The mean ± SDs (*n* = 6 mice/group) shown are representative of two independent experiments. ^∗^*p* < 0.05, ^∗∗^*p* < 0.01 (InsB vs. ESAT-6-immunized group) and ^#^*p* < 0.05, ^##^*p* < 0.01, ^###^*p* < 0.001 (antigen-immunized groups vs. each antigen-treated MPL-DDA group). To reduce false positives of cytokine-producing populations caused by non-specific staining, fewer than 10 cells in lung and 15 cells in spleen were considered as zero. **(C)** Pie charts represent the mean frequencies of cells coexpressing IFN-γ, TNF-α, or IL-2.

Depending on the outcome of the immunization, we next evaluated whether the Th1 immune response generated by immunization with InsB would continue producing effective multifunctional T cells after challenge with virulent Mtb in the spleen and lung over time. To this end, each immunized group was challenged with Mtb after final immunization. Four and 9 weeks after the Mtb challenge, spleen and lung cells from mice were stimulated with InsB and ESAT-6, respectively. The spleen and lung cells were stained for a multifunctional T cell analysis, and the frequency of bi- or triple-positive CD4^+^ and CD8^+^ T cells was analyzed with flow cytometry. As a result, in the InsB- and ESAT-6-immunized groups, triple- and double (IFN-γ^+^TNF-α^+^IL-2^+^, IFN-γ^+^TNF-α^+^, IFN-γ^+^IL-2^+^, and TNF-α^+^IL-2^+^)-positive CD3^+^CD44^+^CD4^+^ and CD3^+^CD44^+^CD8^+^ T cells were observed in both the lung (Figure 4) and spleen (Supplementary Figure S3) compared to those in the MPL-DDA-control groups (each antigen-stimulated MPL-DDA group) at 4 weeks postchallenge. Although more potent multifunctional T cell populations were observed in the ESAT-6-immunized group, the InsB-immunized group showed similar profiles of cytokines secreting CD4^+^ and CD8^+^ T cells (Figure 4 and Supplementary Figure S3). These aspects were observed up to 9 weeks postinfection (Figure 5 and Supplementary Figure S4). Taken together, the results show that immunization with InsB can produce multifunctional Th1 CD4^+^ T cells, including CD8^+^ T cells, and that these reactions might remain for a long time after virulent Mtb strain infection.

## Discussion

Various approaches have been proposed and explored for potential use in TB control. Particularly, prophylactic vaccines have the potential to be less costly and more efficacious than BCG vaccines. Accumulating evidence for a protective immunological response to Mtb infection has shown the importance of the cellular immune response, especially involving the generation of IFN-γ-producing antigen-specific CD4^+^ T cells (Coler et al., 2009). A critical role of IFN-γ in the control of Mtb infection has also been demonstrated in both animals (Cooper et al., 1993; Flynn et al., 1993) and humans (Jouanguy et al., 1996; Newport et al., 1996). Thus, a general strategy to develop the next generation of TB vaccines is to construct subunit vaccines based on T cell antigens (Xiang et al., 2017). In the current study, our results demonstrated that IFN-γ production (Figure 1) and Th1-type multifunctional T cells (Figure 2 and Supplementary Figure S2) were observed in lung and spleen cells from mice immunized with InsB to a similar extent to that observed after immunization with ESAT-6, indicating that InsB elicited an antigen-specific Th1 polarized immune response as an immunogenic antigen.

The identification of Mtb antigens eliciting antigen-specific IFN-γ–producing CD4^+^ T cell responses during Mtb infection has been approached through a variety of methods, including comparative proteomics using biochemical fractionation of the Mtb-secreted antigen pool, comparative transcriptomic analysis, and *in silico* epitope analysis (Shah et al., 2018). The identification of ESAT-6/Rv3875 (Sorensen et al., 1995) and Mtb10.4/Rv0288 (Skjot et al., 2000) via comparative proteomic analysis in a culture filtrate from Mtb is a successful example. Another successful example is the identification of Mtb9.8/Rv0287 using a human CD4^+^ T cell expression cloning approach (Alderson et al., 2000; Coler et al., 2009). These single antigens were integrated into multiantigenic subunit vaccines and are currently entered into the clinical pipeline for new TB vaccine development (Andersen and Kaufmann, 2014). Although these applications have been attempted in many studies, it may be labor-intensive to identify and characterize a single antigen. In the present study, we first identified InsB to be uniquely present in the genome of Mtb K, which is the most prevalent clinical isolate in Korea, by taking advantage of a recent advance in comparative genomic analysis. In particular, InsB has been proven to stimulate the immune response and to have an additive effect to that of ESAT-6 (Park et al., 2014). In addition, InsB showed a comparable antigen-specific Th1-type T-cell response to that induced by ESAT-6, and its immunization demonstrated a remarkable protection efficacy against Mtb K challenge to a similar extent to that with ESAT-6.

In fact, based on its immunodiagnostic potency and protective effectiveness, ESAT-6 is considered the strongest vaccine candidate. ESAT-6 is located in RD1 of the Mtb genome, which is absent in *M. bovis* BCG, and potent T cell antigens with low molecular weights were first identified from a short-term culture filtrate of Mtb (Andersen et al., 1995). Along with the effectors of ESX-1, the antigens encoded in ESX-3 and ESX-5 are also potent inducers of CD4^+^ and CD8^+^ T cells (Brodin et al., 2006; Hoang et al., 2013). These investigations prompted immunological studies of other Esx proteins to determine whether they could display immunogenic qualities (Brodin et al., 2004). These other ESAT-6 family proteins have also received much attention recently with respect to understanding Mtb pathogenesis and characterizing their role in TB vaccine development (Skjot et al., 2002; Homolka et al., 2010). Thus, we identified the Mtb K-specific ESAT-6-like protein InsB and evaluated its protective efficacy against Mtb.

Jones et al. (2010) recently demonstrated similar levels of immunogenicity of the other Esx proteins produced outside the ESX-1 to ESX-5 loci to those of Esx proteins from the ESX-1 to ESX-5 loci. Interestingly, a comparative analysis of Esx genes from clinical isolates of Mtb revealed evidence of gene conversion and epitope variation (Uplekar et al., 2011), particularly among the Mtb9.9 subfamily proteins. Strikingly, even single-amino acid residue differences in the epitope sequences among Mtb9.9 subfamily proteins, which share a high degree of amino acid sequence similarity (93–98%), altered the responder frequencies to individual antigens. In addition, heterogeneous human T cell responses to the Mtb9.9 subfamily proteins were reported (Alderson et al., 2000; Hur et al., 2016). In the present study, we confirmed that there was no cross-reactivity between ESAT-6 and InsB in the induction of cellular and humoral immune responses after each protein immunization (data not shown), which agrees with the results from our previous study (Park et al., 2014).

We finally evaluated the protective efficacy of InsB in a preclinical animal model setting against Mtb K. Interestingly, InsB immunization displayed a similar long-term protective efficacy (up to 9 weeks postinfection) to that of ESAT-6 immunization after challenge with Mtb K based on assessments of lung pathology (Figure 3A) and the reduced bacterial burden (Figure 3B). Although the definitive protective correlates have not been fully elucidated, particularly in humans, the multifunctional abilities of Th1-type T cells were recently shown to be associated with protection against Mtb infection in mouse models (Flynn and Bloom, 1996; Flynn, 2004; Forbes et al., 2008; Karp et al., 2015; Coler et al., 2018). In addition, a continuous decrease in the multifunctionality of T cells corresponds with a decrease in protection during Mtb infection in a mouse model (Nandakumar et al., 2014). We thus detected the maintenance of CD4^+^ T cells capable of producing double- or triple-positive (coexpressing IFN-γ, TNF, and IL-2) Th1 cells via immunization with InsB to a similar extent to that of the multifunctionalities induced by ESAT-6 immunization (Figure 2, 4, 5), suggesting that InsB can be used as a potential vaccine candidate exhibiting immune-stimulatory properties.

In summary, as demonstrated in a murine model, InsB immunization induces strong immunogenicity and has the ability to induce a robust and durable antigen-specific multifunctional Th1-type T cell memory response that confers protective immunity and significant protection against Mtb K, similarly to the effects induced by ESAT-6. These results indicate that InsB is a potential antigen target for the development of future multiantigenic vaccines against Mtb, especially against highly virulent Mtb Beijing strains. However, the evaluation of vaccine efficacy against laboratory-adapted Mtb strains cannot fully reflect the clinical situation (McShane and Williams, 2014; Henao-Tamayo et al., 2015). The Mtb Beijing family, including the K strain, shows a notably higher prevalence and is more frequently isolated in comparison to other Mtb strains (Kang et al., 2010). Therefore, to develop broad-spectrum subunit vaccines against TB, InsB should be further evaluated against non-Beijing Mtb strains as well. In addition, further evaluation of InsB as a booster of the BCG vaccine or in combination with other Esx proteins such as ESAT-6 or TB10.4 to boost additional protective immunity will guarantee the usefulness of InsB as a vaccine antigen.

## Author Contributions

WK, HK, and KK performed the experiments and analyzed the data. S-NC provided tools and critical assistance with experimental procedures. SS and WK designed the experiments, interpreted the data, and wrote the manuscript.

## Conflict of Interest Statement

The authors declare that the research was conducted in the absence of any commercial or financial relationships that could be construed as a potential conflict of interest.

## Footnotes

1 https://blast.ncbi.nlm.nih.gov
