# Supplementary material for: Immunogenicity and Vaccine Potential of InsB, an ESAT-6-Like Antigen Identified in the Highly Virulent Mycobacterium tuberculosis Beijing K Strain
Source: Front Microbiol. 2019 Feb 12;10:220. doi: 10.3389/fmicb.2019.00220 (PMC6379281; doi:10.3389/fmicb.2019.00220)
Supplement: Supplementary file 1 [file Data_Sheet_1.docx]

***Supplementary Material***

**Immunogenicity and Vaccine Potential of InsB, an ESAT-6-like Antigen Identified in the Highly Virulent *Mycobacterium tuberculosis* Beijing K Strain**

Woo Sik Kim^1, 2*^, Hongmin Kim^1*^, Kee Woong Kwon^1*^, Sang Nae Cho^1^, Sung Jae Shin^1†^

^1^Department of Microbiology, Institute for Immunology and Immunological Disease, Brain Korea 21 PLUS Project for Medical Science, Yonsei University College of Medicine, Seoul, South Korea, ^2^Advanced Radiation Technology Institute, Korea Atomic Energy Research Institute, Jeongeup, South Korea

^*^ These authors contributed equally to the work.

^†^Correspondence:

Sung Jae Shin

E-mail address: sjshin@yuhs.ac

Running title: Evaluation of InsB vaccine potential

**
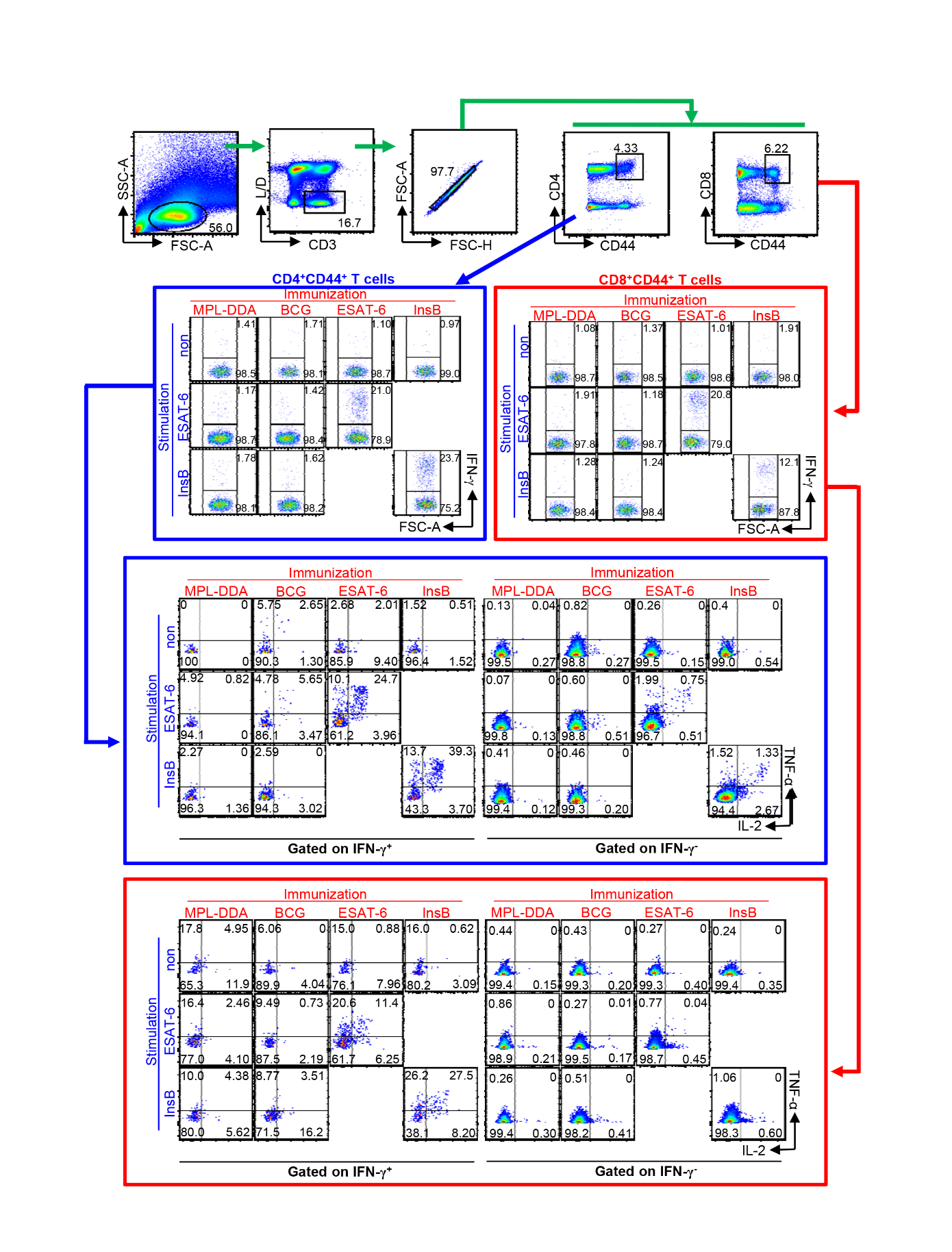
**

**Supplementary Figure 1.** **Flow cytometry analysis of multifunctional T cells.** Spleen and lung cell suspensions were analyzed by multiparameter flow cytometry for cytokine production, and data were collected on a FACSVerse flow cytometer with subsequent analysis using FlowJo software. To analyze cytokine-producing T cells, an inclusion gate was first drawn based on the cell size and granularity. Subsequently, the CD3^+^ lymphocytes were gated, and the dead cell population was excluded using the Live/Dead viability dye. Single-cell populations were then gated to exclude doublets and larger cell aggregates by removing equivalent forward scatter-height and forward scatter-area values. The activated T cells were further gated to obtain CD4^+^CD44^+^ and CD8^+^CD44^+^ cells and thus limit the analysis of cytokine-producing cells to T lymphocytes. To distinguish multifunctional T cell subsets, gates indicating positive staining for each cytokine in unstimulated or stimulated cell populations were delineated using the isotype controls to determine the background staining. These data were then used to delimit the production of each of the three cytokines by every gated CD44^+^ T cell, and the proportion of total events constituting each cytokine-producing subset was determined. Values were converted to absolute numbers by multiplying these proportions by the total cell yield obtained from the lungs of each animal. These gating strategies are representatively described using splenocytes from the immunized mice. The cells were analyzed with a FACSVerse flow cytometer using FlowJo software. This FACSVerse flow cytometer is composed of 3-laser system, providing 8-color configuration in maximum. Emitted light excited by the violet laser (405 nm) is filtered through a 528/45 filter (corresponding to a 500LP mirror) and 488/45 filter (488/45 mirror). Light excited by the blue laser (488 nm) is filtered through a 783/56 filter (725LP mirror), 700/54 filter (665 LP mirror), 586/42 filter (560 LP mirror), and 527/32 filter (507 LP mirror). Light excited by the red laser (640 nm) is filtered through a 783/56 filter (752 LP mirror) and 660/10 filter (660/10 mirror).

**
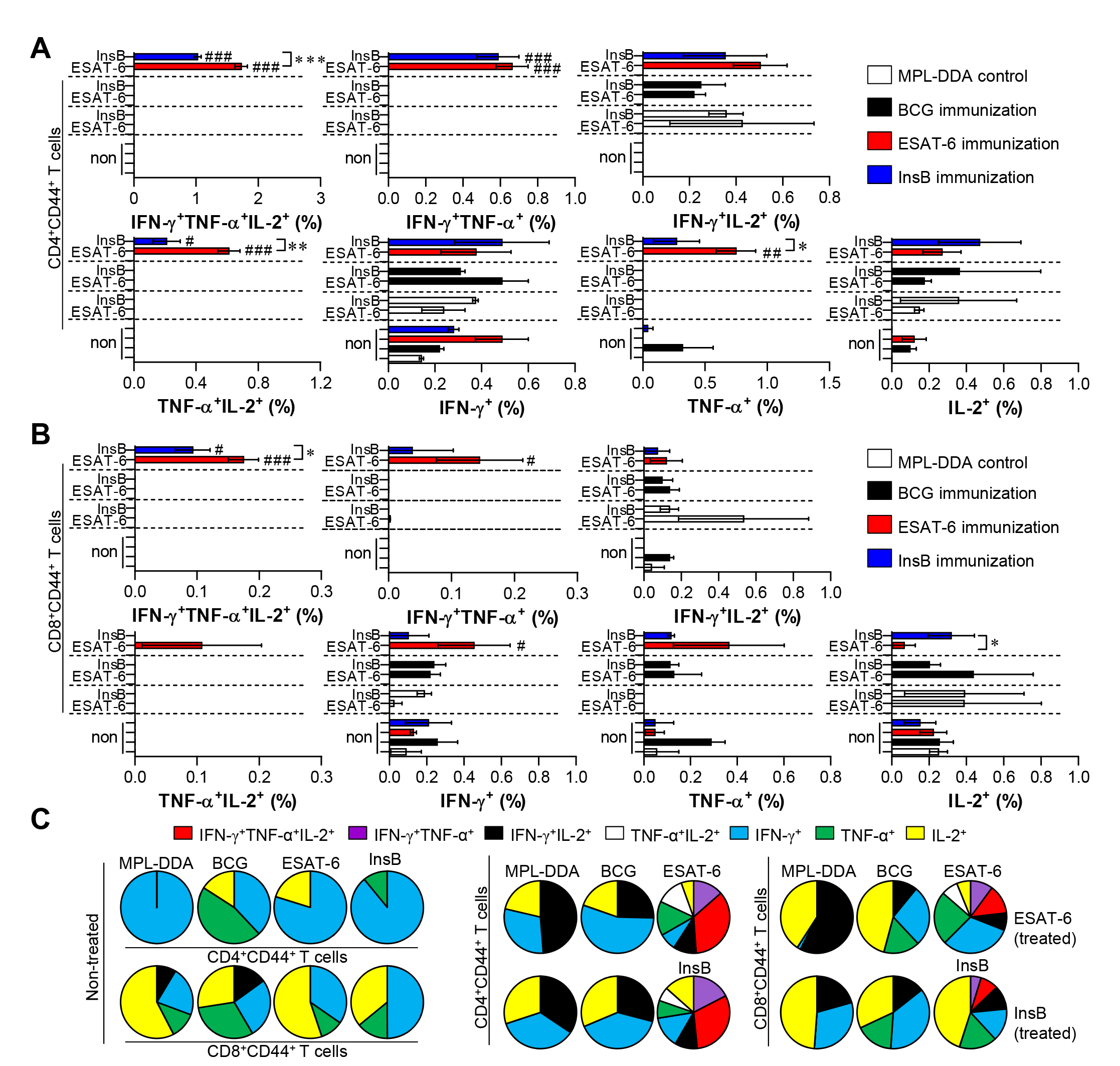
**

**Supplementary Figure 2.** **Induction of antigen-specific multifunctional T cells in the spleen of ESAT-6- and InsB-immunized mice.** At 3 weeks after the last immunization, splenocytes from adjuvant (MPL-DDA)-, BCG-, ESAT-6-, or InsB-immunized mice (*n* = 6 mice/group) were stimulated *in vitro* with no antigen, with ESAT-6, or with InsB in the presence of GolgiStop for 12 h at 37°C. The percentage of antigen-specific CD3^+^CD4^+^CD44^+^ (**A**) and CD3^+^CD8^+^CD44^+^ (**B**) T cells producing IFN-γ, TNF-α, or IL-2 was measured. The frequency of T cells producing each combination of cytokines is shown as the percentage of the specific cell type in the CD3^+^CD4^+^CD44^+^ or CD3^+^CD8^+^CD44^+^ T cell population. The mean ± SDs (*n* = 6 mice/group) shown are representative of two independent experiments. **p* < 0.05, ***p* < 0.01, or ****p* < 0.001 (InsB *vs.* ESAT-6-immunized group) and ^#^*p* < 0.05, ^##^*p* < 0.01, ^###^*p* < 0.001 (antigen-immunized groups *vs.* each antigen-treated MPL-DDA group). To reduce false positives of cytokine-producing populations caused by non-specific staining, fewer than 10 cells in lung and 15 cells in spleen were considered as zero. (**C**) Pie charts represent the mean frequencies of cells coexpressing IFN-γ, TNF-α, or IL-2.

**
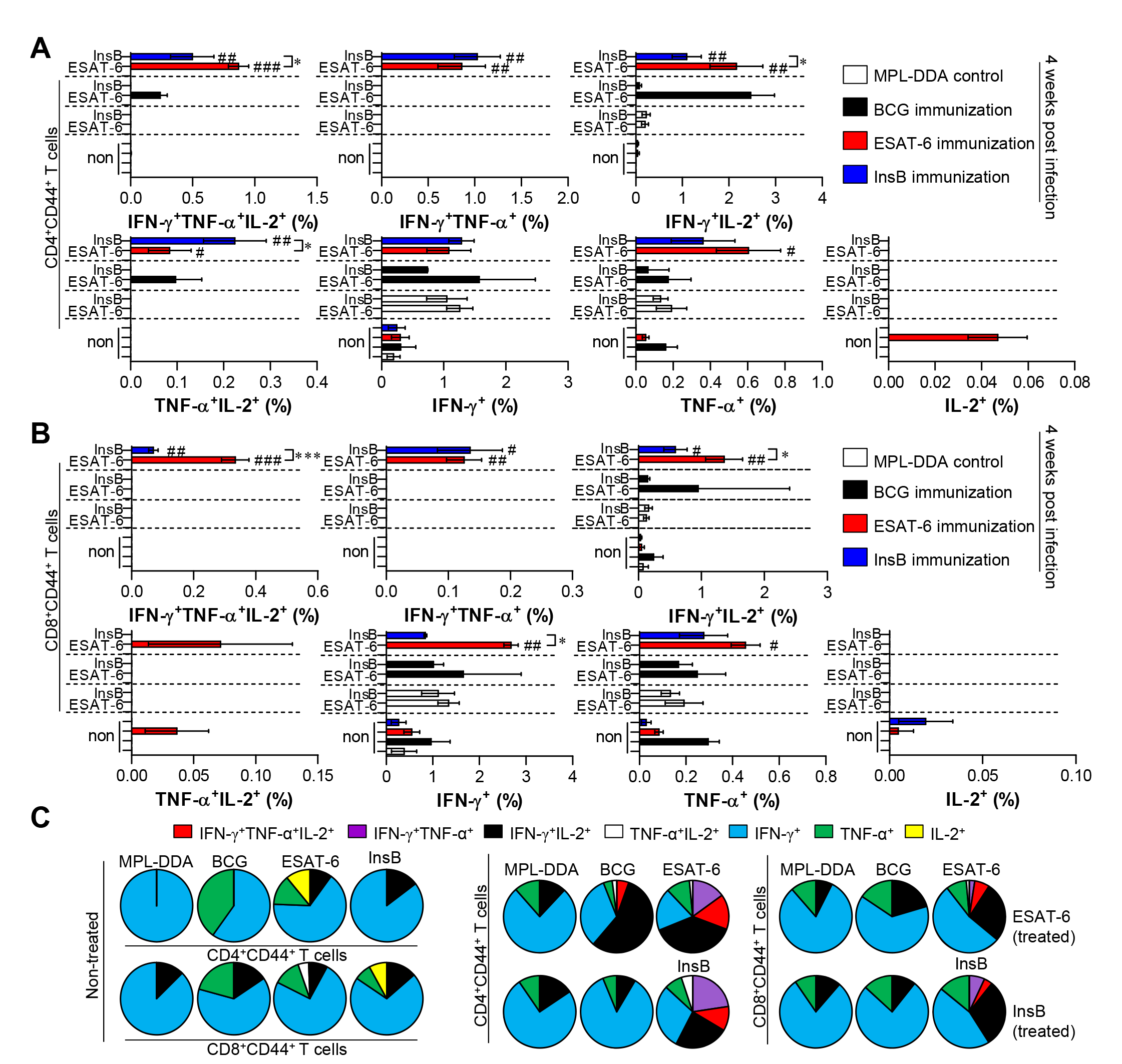
**

**Supplementary Figure 3.** **Induction of antigen-specific multifunctional T cells in the spleen of ESAT-6- and InsB-immunized mice at 4 weeks postinfection.** Four weeks postinfection, splenocytes from adjuvant (MPL-DDA)-, BCG-, ESAT-6-, or InsB-immunized mice (*n* = 6 mice/group) were stimulated *in vitro* with no antigen, with ESAT-6, or with InsB in the presence of GolgiStop for 12 h at 37°C. The percentage of antigen-specific CD3^+^CD4^+^CD44^+^ (**A**) and CD3^+^CD8^+^CD44^+^ (**B**) T cells producing IFN-γ, TNF-α, or IL-2 were analyzed using flow cytometry. The frequency of T cells producing each combination of cytokines is presented as the percentage of the specific cell type in the CD3^+^CD4^+^CD44^+^ or CD3^+^CD8^+^CD44^+^ T cell population. The mean ± SDs (*n* = 6 mice/group) shown are representative of two independent experiments. **p* < 0.05, or ****p* < 0.001 (InsB *vs.* ESAT-6-immunized group) and ^#^*p* < 0.05, ^##^*p* < 0.01, ^###^*p* < 0.001 (antigen-immunized groups *vs.* each antigen-treated MPL-DDA group). To reduce false positives of cytokine-producing populations caused by non-specific staining, fewer than 10 cells in lung and 15 cells in spleen were considered as zero. (**C**) Pie charts represent the mean frequencies of cells coexpressing IFN-γ, TNF-α, or IL-2.

**
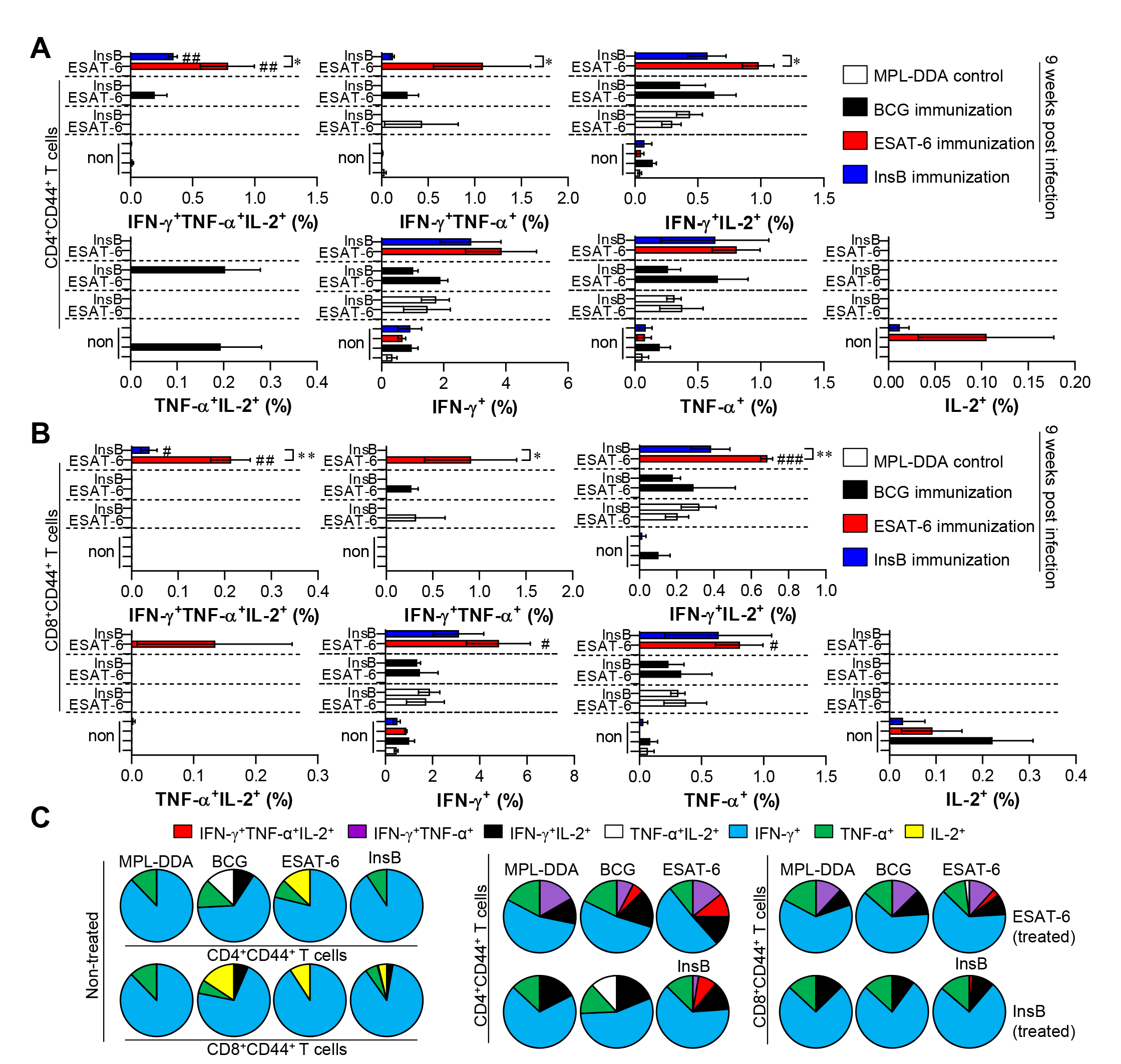
**

**Supplementary Figure 4.** **Induction of antigen-specific multifunctional T cells in the spleen of ESAT-6- and InsB-immunized mice at 9 weeks postinfection.** Nine weeks postinfection, splenocytes from adjuvant (MPL-DDA)-, BCG-, ESAT-6-, or InsB-immunized mice (*n* = 6 mice/group) were stimulated *in vitro* with no antigen, with ESAT-6, or with InsB in the presence of GolgiStop for 12 h at 37°C. The percentage of antigen-specific CD3^+^CD4^+^CD44^+^ (**A**) and CD3^+^CD8^+^CD44^+^ (**B**) T cells producing IFN-γ, TNF-α, or IL-2 was analyzed using flow cytometry. The frequency of T cells producing each combination of cytokines is shown as the percentage of the specific cell type in the CD3^+^CD4^+^CD44^+^ or CD3^+^CD8^+^CD44^+^ T cell population. The mean ± SDs (*n* = 6 mice/group) shown are representative of two independent experiments. **p* < 0.05, or ***p* < 0.01 (InsB *vs.* ESAT-6-immunized group) and ^#^*p* < 0.05, ^##^*p* < 0.01, ^###^*p* < 0.001 (antigen-immunized groups *vs.* each antigen-treated MPL-DDA group). To reduce false positives of cytokine-producing populations caused by non-specific staining, fewer than 10 cells in lung and 15 cells in spleen were considered as zero. (**C**) Pie charts represent the mean frequencies of cells coexpressing IFN-γ, TNF-α, or IL-2.
